# Multi frequency multi bit amplitude modulation of spoof surface plasmon polaritons by schottky diode bridged interdigital SRRs

**DOI:** 10.1038/s41598-021-98846-4

**Published:** 2021-09-28

**Authors:** Haotian Ling, Baoqing Zhang, Mingming Feng, Pengfei Qian, Yiming Wang, Qingpu Wang, Yifei Zhang, Aimin Song

**Affiliations:** 1 Shandong Technology Center of Nanodevices and Integration and School of Microelectronics, Shandong University, Jinan, 250100 China; 2 School of Electrical and Electronic Engineering, University of Manchester, Manchester, M13 9PL UK

**Keywords:** Nanophotonics and plasmonics, Metamaterials, Electrical and electronic engineering

## Abstract

Multi-frequency multi-bit programmable amplitude modulation (AM) of spoof surface plasmon polaritons (SPPs) is realized at millimeter wave frequencies with interdigital split-ring resonators (SRRs) and In-Ga-Zn-O (IGZO) Schottky diodes. Periodic SRRs on a metal line guide both SRR mode and spoof SPP mode, the former of which rejects the spoof SPP propagation at the SRR resonant frequencies. To actively modulate the amplitude of spoof SPPs, IGZO Schottky diodes are fabricated in the SRR gaps, which continuously re-configure SRRs to metallic loops by applying bias. Interdigital gaps are designed in SRRs to increase the capacitance, thus red shifting the resonant frequencies, which significantly broadens the operation bandwidth of multi-frequency AM. Thus, cascading different kinds of interdigital SRRs with Schottky diodes enables multi-frequency multi-bit AM programmable. As a demonstration, a dual-frequency device was fabricated and characterized, which achieved significant multi-bit AM from −12.5 to −6.2 dB at 34.7 GHz and from −26 to −8.5 dB at 50 GHz independently and showed programmable capability.

## Introduction

Artificial metallic structures, such as periodic holes and grooves have been reported to mimic surface plasmon polaritons (SPPs) at millimeter wave and terahertz frequencies where metals are regarded as perfect electric conductors^1^. As the low-frequency counterparts of optical SPPs, these structures imitate the same features of strong field confinement and non-diffraction limit, and typically are referred to as “spoof” SPPs^2–4^. Differing from natural SPPs, the dispersion characteristics of spoof SPPs can be designed by changing the geometric dimensions. On account of the abilities to conquer the diffraction limit and enhance the localized electric field, spoof SPPs have inspired many potential applications in sub-wavelength resolution imaging^5,6^, sub-wavelength circuits^7^, photolithography^8^, sensing^9,10^, etc.

Recently, split-ring resonators (SRRs) and complementary SRRs have been investigated to achieve a rejection function of spoof SPPs, such as ultra-wideband and multi-frequency band-stop filters^11–14^. To actively modulate the rejection, varactor diodes and Schottky diodes have been integrated with SRR structures, achieving frequency modulation and amplitude modulation, respectively^15–21^. These devices have been potentially applied to electrically program spoof SPPs for multifunctional applications. The approached of varactor diode were reported below 15 GHz, which may not be suitable for monolithic fabrication and millimeter wave frequencies due to the soldering integration^15–19^. Based on soldered varactor diodes, multi-frequency frequency modulation of spoof SPPs has been demonstrated below 10 GHz^19^. However, multi-frequency amplitude modulation (AM) of spoof SPPs has not been reported so far to the best of the authors’ knowledge.

In this work, we report multi-frequency multi-bit programmable AM of spoof SPPs based on interdigital SRRs and In-Ga-Zn-O (IGZO) Schottky diodes. In the proposed device, interdigital capacitor substitutes for parallel plate capacitor to get larger AM spectral range without sacrificing the AM depth. The SRR-based device has two modes: the fundamental one is SRR resonance, and the second-order one is spoof SPP mode. To dynamically modulate the amplitude, IGZO Schottky diodes are designed in the SRR gaps to gradually short the gap with applied bias, and thus achieve reconfiguration. To verify our design, a dual-frequency AM device was simulated, fabricated, and characterized. By using two separate bias, the transmission of spoof SPPs can be programmably tuned from −12.5 to −6.2 dB at 34.7 GHz and from −26 to −8.5 dB at 50 GHz independently, showing significant AM at millimeter wave frequencies.

### Passive spoof SPP filter with interdigital SRRs

**Figure 1:**
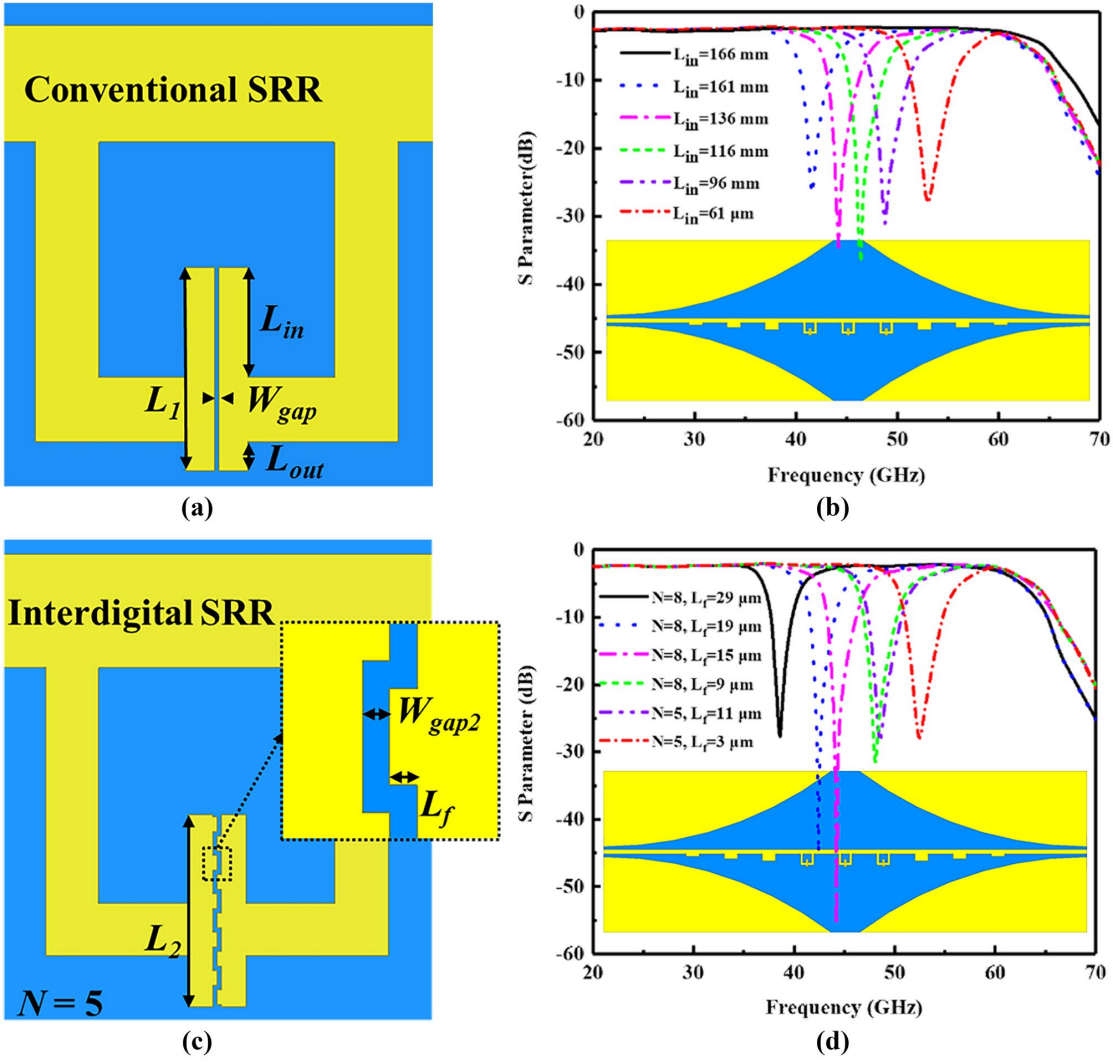
(**a**) Conventional SRR with parallel gap (*L*_*1*_ = 135 μm, *W*_*gap*_ = 3 μm). (**b**) Resonant response of conventional SRR based spoof SPP device (as the inset depicted). (**c**) Interdigital SRR (*L*_*2*_ = 135 μm, *W*_*gap2*_ = 3 μm, *L*_*f*_ = 3 μm, *N* = 5) and zoom-in view of the interdigital gap. (**d**) Resonant response of interdigital SRR based spoof SPP device (as the inset depicted).

Typically, SRRs in spoof SPP band-reject devices use parallel gaps^20,21^, as illustrated in Fig. 1a. For multi-frequency modulation, the gap capacitance needs to be swept by changing gap dimensions, e.g., length. Take the SRRs in our previous paper as an example^21^, whose gap width and length are *W*_*gap*_ = 3 μm, and *L*_*1*_ = 135 μm, respectively. Extending *L*_*out*_ changes the spoof SPP cut-off frequency so that increasing *L*_*in*_ is a better choice. However, as *L*_*in*_ increases, parasitic coupling gets stronger and the Q-factor of SRR resonance decreases significantly. To clarify the impact, 3-D models are built in Ansys High Frequency Structural Simulator (HFSS), and their S-parameters are calculated by using Driven Mode solver. As shown in Fig. 1b, the Q-factor reduces from 171 to 0 as the frequency decreases. When *L*_*in*_ = 166 μm, the capacitive structure contacts the SRR loop, which eliminates the SRR resonance, as the black curve shown in Fig. 1b. In this respect, interdigital capacitors are designed to broaden the operation bandwidth, see Fig. 1c. The SRR has interdigital fingers in the split gap, whose dimensions are *W*_*gap2*_ = 3 μm, *L*_*2*_ = 135 μm, *L*_*f*_ = 3 μm, and finger number is *N* = 5. The resonant responses of the spoof SPP waveguide with different interdigital SRRs are illustrated in Fig. 1d. It can be seen that increasing finger length *L*_*f*_ is an effective way to acquire lower resonant frequencies without sacrificing Q-factor. In addition, introducing more fingers can further reduce the resonant frequency. When *N* = 8 and *L*_*f*_ = 15 μm, the Q-factor is 1470 at 44 GHz; when *N* = 8, *L*_*f*_ = 29 μm, the Q-factor is 110 at 37.4 GHz. Note that the Q-factor is similar to the one without interdigital SRRs at 50 GHz.

**Figure 2:**
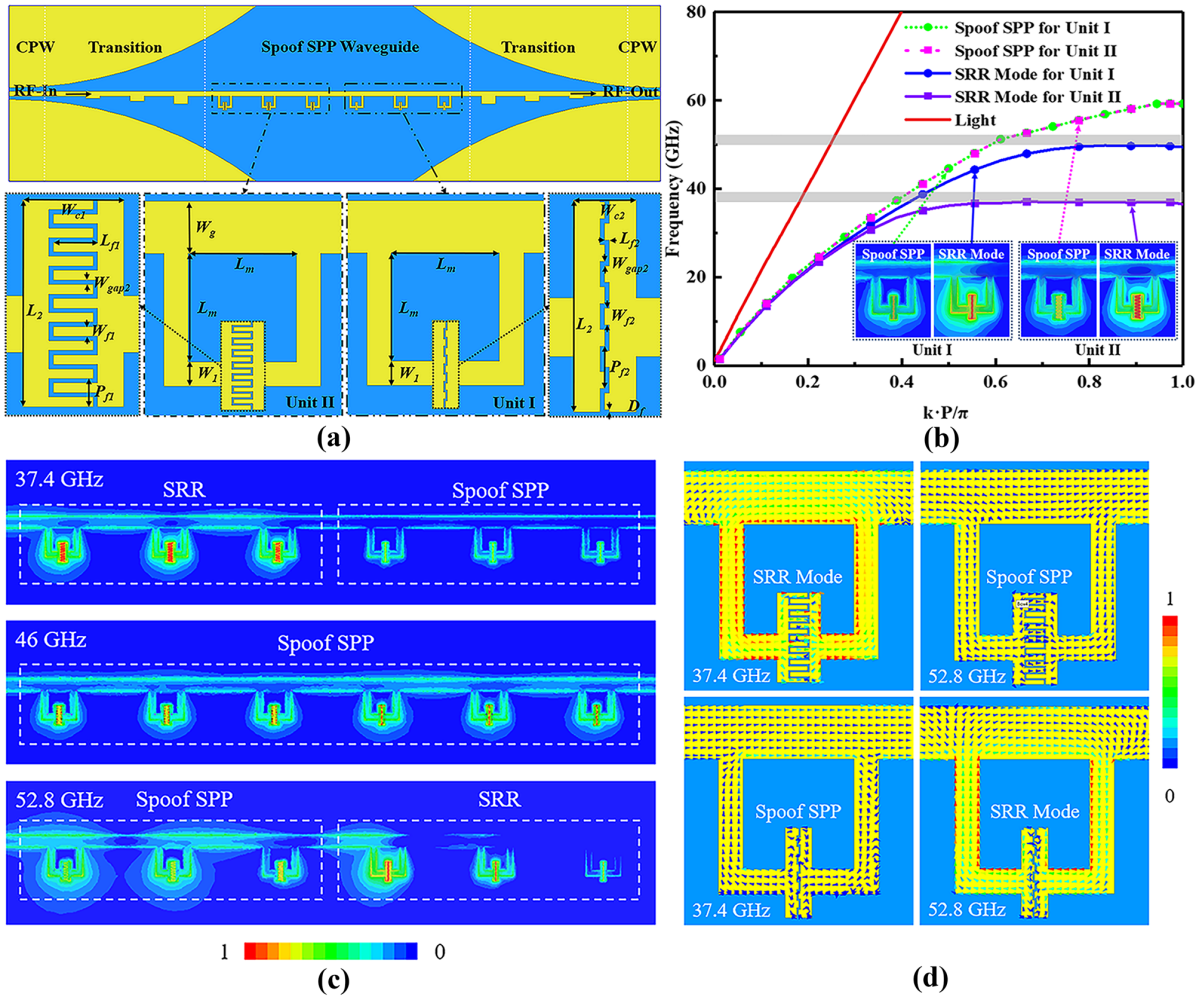
(**a**) Schematics of the two-frequency spoof SPP device with interdigital SRRs (The yellow part is gold, and the blue part is Si), whose dimensions are *W*_*g*_ = 80 μm, *L*_*m*_ = 166 μm, *W*_*1*_ = 37 μm, *L*_*2*_ = 135 μm, *W*_*c1*_ = 66 μm, *L*_*f1*_ = 29 μm, *W*_*gap2*_ = 3 μm, *W*_*f1*_ = 6.2 μm, *P*_*f1*_ = 29 μm, *W*_*c2*_ = 40 μm, *L*_*f2*_ = 3 μm, *W*_*gap2*_ = 3 μm, *W*_*f2*_ = 10.5 μm, *P*_*f2*_ = 27 μm, *D*_*f*_ = 1.5 μm. (**b**) Dispersion diagram of the spoof SPP units. The grey rectangles are rejection bands. The inset shows the spoof SPP and SRR modes of the two spoof SPP units. (**c**) E-field distributions of the spoof SPP device with in-series interdigital SRRs. (**d**) Surface current density distributions the two spoof SPP units.

For multi-frequency AM, we employ two sets of interdigital SRRs, whose resonant frequencies are 37.4 and 52.8 GHz, respectively, as shown in Fig. 2. Their specific dimensions can be obtained from the option of Fig. 2a. The proposed device is designed on silicon substrate, consisting of CPWs for feeding, CPW-to-spoof SPP waveguide transitions for momentum matching and interdigital SRRs. The whole device has a total length of 11.13 mm (about 1.38 wavelength at 37.4 GHz), which is much smaller than the reported multi-frequency spoof SPPs^11–14,19^. The dispersion curves of the spoof SPP units I and II with different SRRs are shown in Fig. 2b, which is simulated by using HFSS Eigen Mode Solver. The pitch of the in-series SRRs is 0.75 mm. Each spoof SPP unit has two modes: the first is SRR resonant mode^21^, as the dotted solid lines shown, and the latter is spoof SPP mode, as the dotted dash lines shown. It can be clearly seen that all four dispersion curves gradually separate from the curve of light, and then asymptotically arrive at a cut-off frequency. Due to the different interdigital gaps, the two SRRs in Fig. 2a have different resonant frequencies, i.e., 37.4 and 52.8 GHz. The grey parts in Fig. 2b elucidate the rejection bands for spoof SPPs due to the SRR resonances^12^. At the non-resonant frequencies, these two SRRs works as the conventional metallic rectangles to guide spoof SPPs^20^, and share the same cut-off frequency of 62 GHz, which corresponds to the dotted dash lines in Fig. 2b. Due to the same profile dimensions and period, the dispersion curves of the two spoof SPP units overlap, which can be explained using Eq. (1) in Ref.^22^.

To further illustrate the discrepancy between spoof SPP and SRR modes, the E-field distributions of the spoof SPP device with in-series interdigital SRRs are shown in Fig. 2c. At the resonant frequencies, the SRR modes have strong E-field confinement in their split gaps, which significantly attenuates wave propagation. At the non-resonant frequencies, the spoof SPP modes have relatively uniform field distributions in the split rings. From Fig. 2d, we can clearly observe various surface current density distributions for the resonant SRR modes and non-resonant spoof SPP modes.

**Figure 3:**
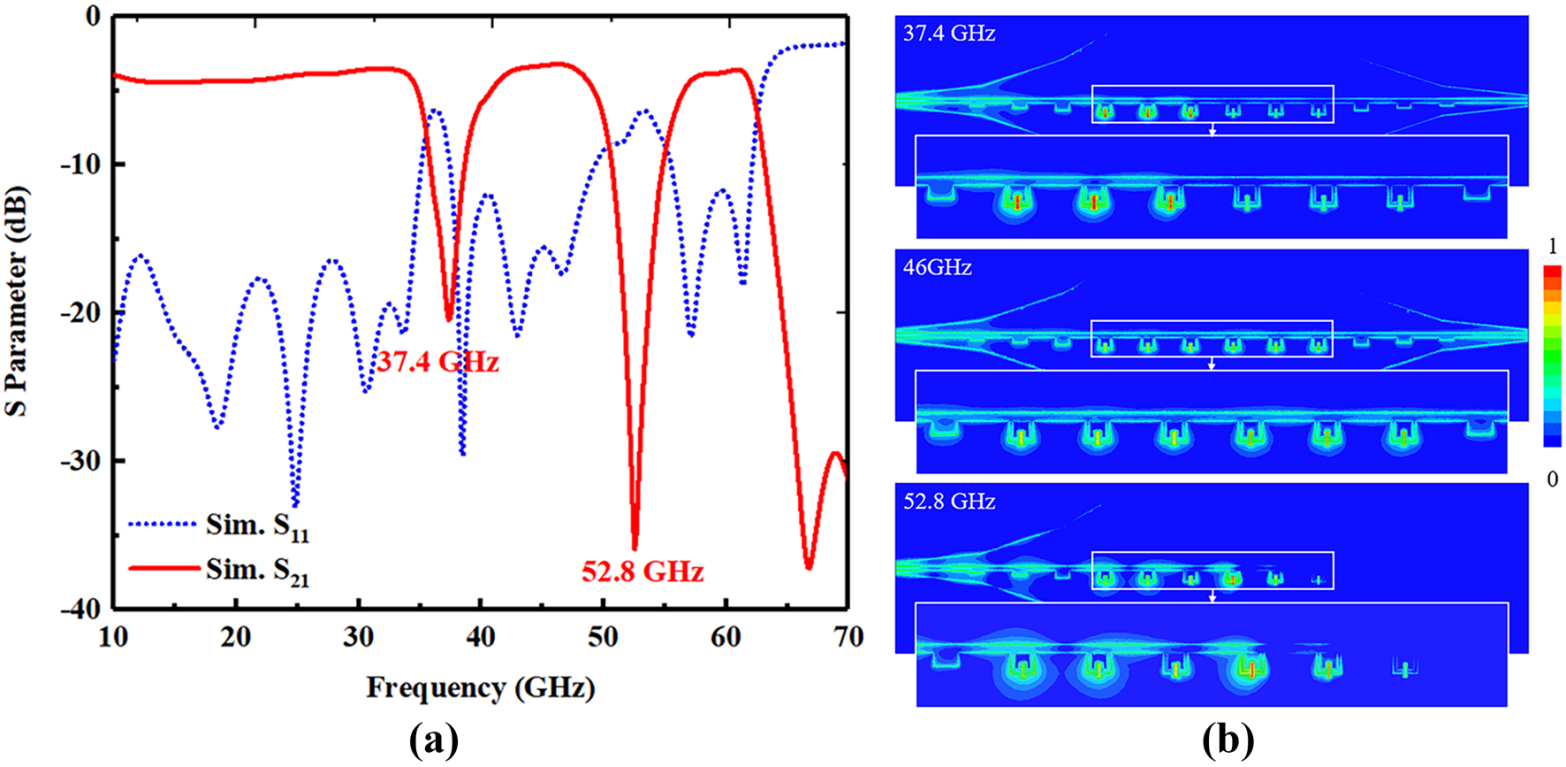
(**a**) Simulated S-parameters of the passive spoof SPP device. (**b**) E-field distribution of the device.

The simulated transmission and reflection of the passive devices are illustrated in Fig. 3a. The SRR resonant frequencies are 37.4 and 52.8 GHz, respectively, which fits dispersion curves in Fig. 2b very well. Figure 3b shows the E-field distribution of the passive device. At the resonant frequencies, e.g. 37.4 and 52.8 GHz, spoof SPPs are strongly reflected due to the SRR resonance. At the non-resonant frequencies, e.g. 46 GHz, spoof SPPs propagate to the signal output terminal with little loss.

### Dual-frequency multi-bit amplitude modulation

**Figure 4:**
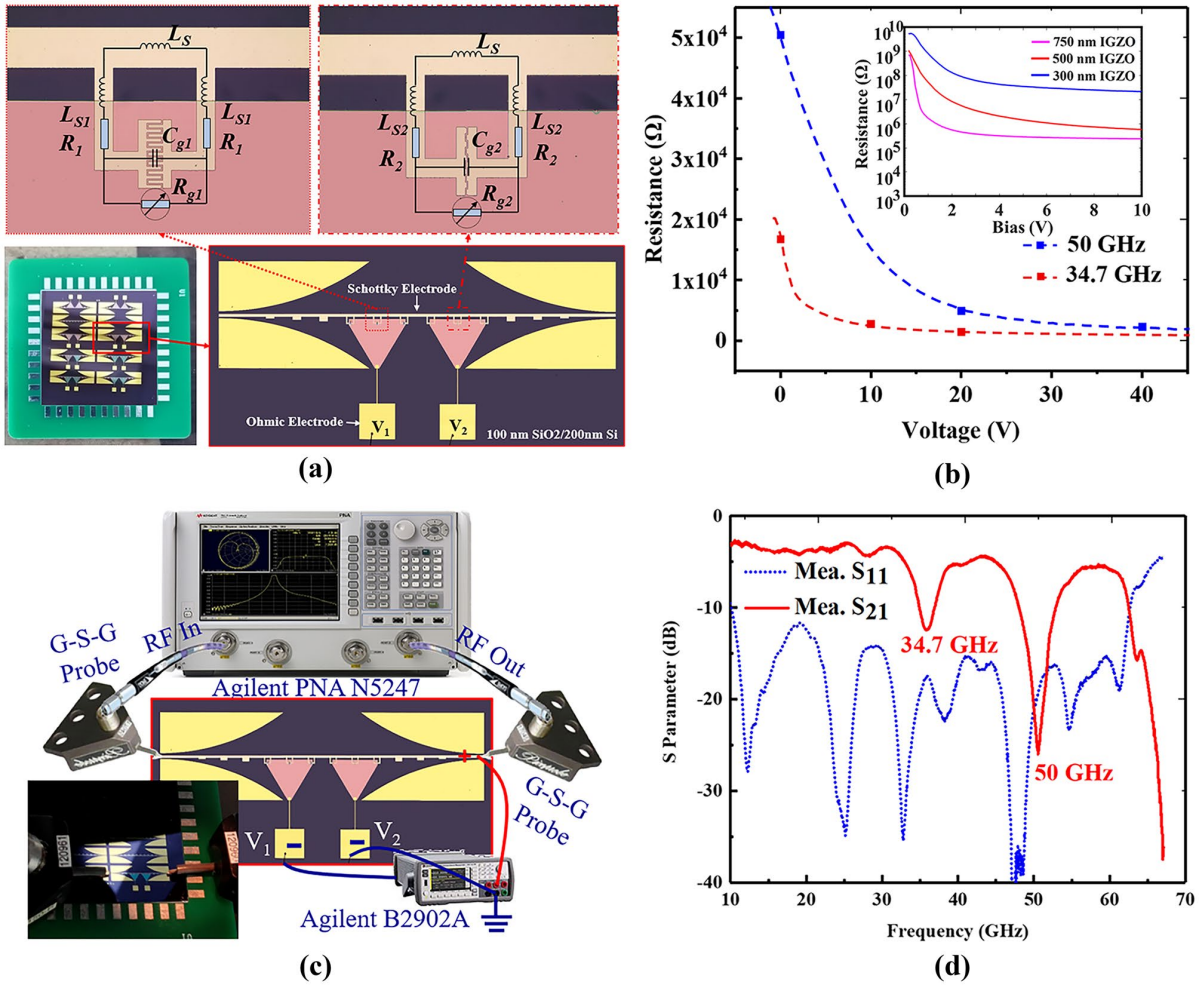
(**a**) Fabricated tunable two-frequency spoof SPP device and equivalent circuit models for the two spoof SPP units. (**b**) IGZO resistance within the SRR gap as a function of voltage at 34.7 GHz and 50 GHz, respectively. The inset shows the resistance–voltage curves of the Schottky diodes with various IGZO thickness. (**c**) System set-up with Agilent PNA N5247 and SMU B2902A. The inset shows the device under test. (**d**) Measured S-parameters of the fabricated device.

The fabricated two-frequency AM device is shown in Fig. 4a, which is made with standard photolithography and vacuum evaporation technology. The dark area is 200-μm thick silicon substrate with a resistivity of 10,000 Ω·mm and a 100-nm SiO_2_ insulator. The pink area is a 500-nm amorphous IGZO film with an electron mobility of 10–50 cm^2^/Vs, which was deposited by RF sputtering at room temperature. In addition to the merits of low-temperature fabrication process, large area, high yield, and low cost, amorphous IGZO has found attractive prospects in flexible applications and large-area industrial fabrication^23–25^. The transmission line with interdigital SRRs, i.e., Schottky electrode, is composed of 10 nm Ti/300 nm Au/ 50 nm Pd. Prior to IGZO deposition, the Pd film was treated with oxygen plasma to form Schottky junction at the interface with IGZO film. The square Ohmic electrodes composed of 10 nm Ti/300 nm Au are bonded to the printed circuit board (PCB) with gold wire. Thus, the left three SRRs are controlled by the input voltage *V*_*1*_ and the right three SRRs are controlled by the input voltage *V*_*2*_.

**Figure 5:**
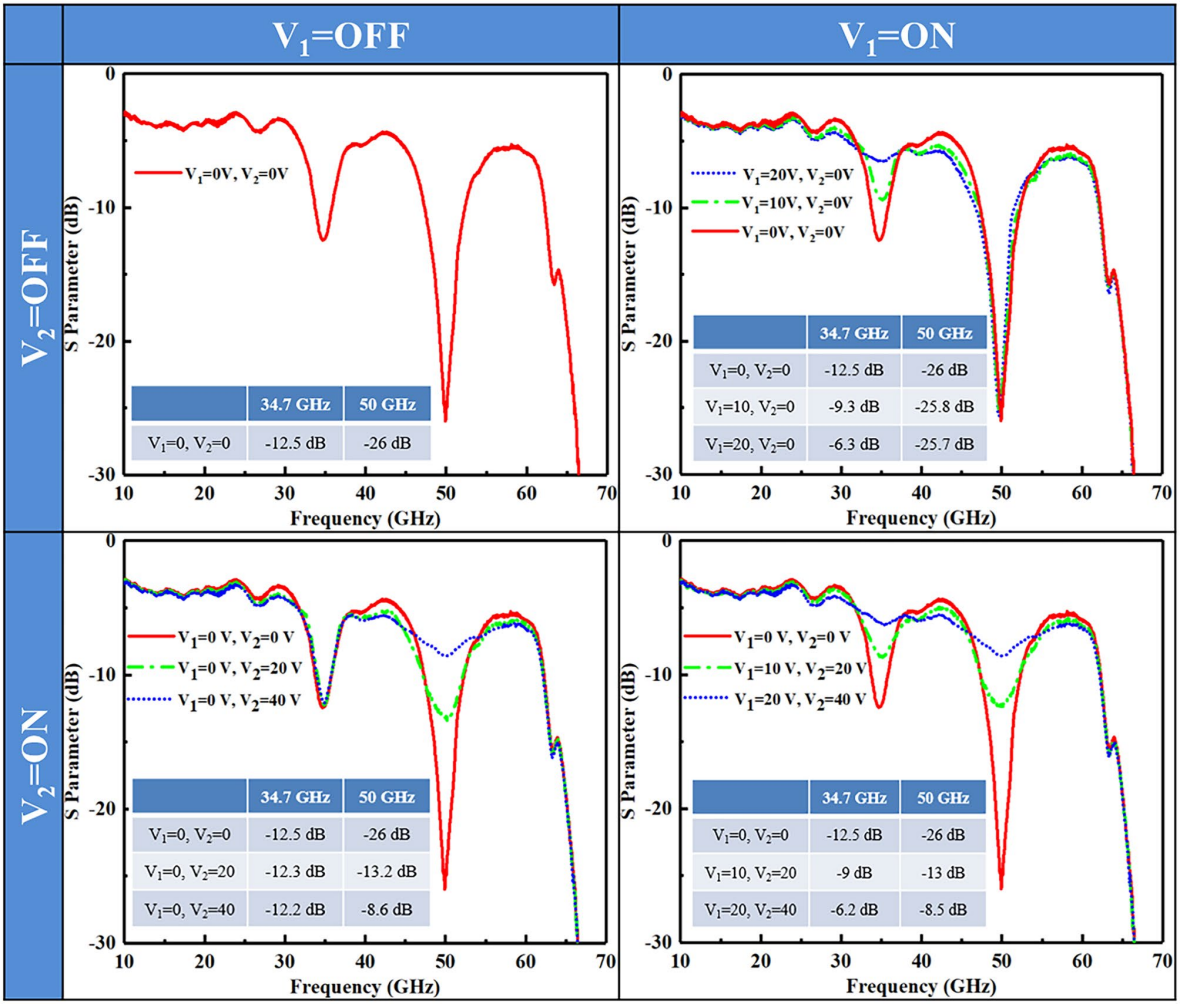
Dual-frequency multi-bit AM of spoof SPPs with individual bias. The inset shows the transmission values under various bias at 34.7 and 50 GHz.

To better show the mechanism, the equivalent circuit model of the two spoof SPP units with an IGZO Schottky diode is shown in Fig. 4a. The SRR can be regarded as *RLC* circuit^26^. *Ls* is the inductance of the coincidence region of SRR and transmission line, *R*_*1*_ and *R*_*2*_ are the resistances of the ring, *L*_*S1*_ and *L*_*S2*_ are inductances of the ring and *C*_*g1*_ and *C*_*g2*_ are the capacitances of the interdigital capacitors. *R*_*g1*_ and *R*_*g2*_ are variable attenuation due to the substrate free carries absorption within the SRR gap^20^. The parameters of circuit components in Fig. 4a were calculated using the methods reported in Ref.^26^, as listed in Table 1. The resistance–voltage curves of Schottky diodes with various IGZO thickness is illustrated as the inset picture in Fig. 4b. The channel length and width are 3 and 40 μm, respectively. Note that the channel resistance reduces as the film thickness enlarges. When the DC bias is zero, the phenomenon of the IGZO film depletion in the gap is equivalent to a large resistance, so that a strong rejection is induced with SRR resonance. When the bias increases, the free carriers with increasing density gradually short out the capacitor, thus the attenuation reduces. The corresponding relation between resistance and bias is illustrated in Fig. 4b. *R*_*g1*_ is 16,800 Ω, 2780 Ω, and 1470 Ω at 34.7 GHz with *V*_*1*_ = 0, 10, 20 V, respectively, and *R*_*g2*_ is 50,500 Ω, 4960 Ω, and 2300 Ω at 50 GHz with *V*_*2*_ = 0, 20, 40 V, respectively, which corresponds to the multi-bit modulation in Fig. 5.

**Table 1:** Equivalent circuit component value of the proposed SRR at the operational frequencies.

34.7 GHz		50 GHz	
*L*_*S*_	0.116 nH	*L*_*S*_	0.116 nH
*L*_*S1*_	0.14 nH	*L*_*S2*_	0.15 nH
*R*_*1*_	0.22 Ω	*R*_*2*_	0.16 Ω
*C*_*g1*_	50.6 fF	*C*_*g2*_	50.6 fF

Agilent programmable network analyzer (PNA) N5247 and dual channel source measure unit (SMU) B2902A was employed to characterize the proposed device, and the system set-up is illustrated in Fig. 4(c). The measured S-parameter of the fabricated spoof SPPs at zero forward bias are illustrated in Fig. 4(d), showing a good agreement with the simulated data. It can be seen that the proposed device has two stop band at 34.7 GHz and 50 GHz, respectively. And its propagation attenuation is less than 5.5 dB out of the stop band. Compared with simulated results, the central frequencies of both resonant responses have a red shift of about 2.7 GHz due to the dielectric constant of a-IGZO^23^.

The transmission modulation of the fabricated device with different bias are illustrated in Fig. 5, showing large AM depth at different frequencies and good spectral stability. When *V*_*1*_ is on and *V*_*2*_ is off, the transmission at 34.7 GHz can be continuously tuned from −12.5 to −6.3 dB with increasing input voltage *V*_*1*_, as shown in the top right figure. When *V*_*1*_ is off and *V*_*2*_ is on, the transmission at 50 GHz can be tuned from −26 to −8.6 dB with a forward bias up to 40 V, as shown in the bottom left figure. When *V*_*1*_ and *V*_*2*_ are open at the same time, the two-frequency device achieves significant AM of from −12.5 to −6.2 dB at 34.7 GHz and from −26 to −8.5 dB at 50 GHz independently, as shown in the bottom right figure. It should be noted that the resonant deeps show little spectral shift within a large range of the applied bias. Additionally, 3-bit AM has been demonstrated at 34.7 and 50 GHz independently and simultaneously, as illustrated in Fig. 5. The spoof SPP transmission is −12.5 dB, −9 dB, and −6.2 dB at 34.7 GHz with *V*_*1*_ = 0, 10, 20 V, respectively, and is −26 dB, −13 dB, and −8.5 dB at 50 GHz with *V*_*2*_ = 0, 20, 40 V, respectively. With more different SRRs and more independent bias, multi-frequency AM of spoof SPPs can be achieved with multi-bit modulation from 34.7 to 50 GHz, which enables a novel approach for programmable AM within a large bandwidth.

## Conclusion

We proposed a programmable approach for multi-frequency multi-bit AM of spoof SPPs with interdigital SRRs by using IGZO Schottky diodes. Interdigital SRRs are proposed to obtain large operation bandwidth of from 37.4 to 52.8 GHz with minimized parasitic coupling and high Q-factor. Schottky diode fabricated in the SRR gap reconfigures the SRRs to metallic loops, which enables significant AM of from −12.5 to −6.2 dB at 34.7 GHz and from −26 to −8.5 dB at 50 GHz, respectively. Based on the interdigital SRRs and IGZO Schottky diodes, a dual-frequency device with cascaded SRRs was designed and fabricated, achieving 3-bit AM at 34.7 and 50 GHz with programmable bias. The proposed method provides a novel insight into multi-frequency multi-bit programmable modulation of spoof SPPs at microwave and THz regime.
